# Trend of Urban-Rural Disparities in Hospice Utilization in Taiwan

**DOI:** 10.1371/journal.pone.0062492

**Published:** 2013-04-26

**Authors:** Yi-Hsuan Lin, Yi-Chun Chen, Yen-Han Tseng, Ming-Hwai Lin, Shinn-Jang Hwang, Tzeng-Ji Chen, Li-Fang Chou

**Affiliations:** 1 Department of Family Medicine, Kaohsiung Veterans General Hospital Pingtung Branch, Pingtung, Taiwan; 2 Department of Family Medicine, Taitung Veterans Hospital, Taitung, Taiwan; 3 Respiratory Therapy Department, Taipei Veterans General Hospital, Taipei, Taiwan; 4 Department of Family Medicine, Taipei Veterans General Hospital, Taipei, Taiwan; 5 School of Medicine, National Yang-Ming University, Taipei, Taiwan; 6 Department of Public Finance, National Chengchi University, Taipei, Taiwan; Indiana University, United States of America

## Abstract

**Aims:**

The palliative care has spread rapidly worldwide in the recent two decades. The development of hospice services in rural areas usually lags behind that in urban areas. The aim of our study was to investigate whether the urban-rural disparity widens in a country with a hospital-based hospice system.

**Methods:**

From the nationwide claims database within the National Health Insurance in Taiwan, admissions to hospices from 2000 to 2006 were identified. Hospices and patients in each year were analyzed according to geographic location and residence.

**Results:**

A total of 26,292 cancer patients had been admitted to hospices. The proportion of rural patients to all patients increased with time from 17.8% in 2000 to 25.7% in 2006. Although the numbers of beds and the utilizations in both urban and rural hospices expanded rapidly, the increasing trend in rural areas was more marked than that in urban areas. However, still two-thirds (898/1,357) of rural patients were admitted to urban hospices in 2006.

**Conclusions:**

The gap of hospice utilizations between urban and rural areas in Taiwan did not widen with time. There was room for improvement in sufficient supply of rural hospices or efficient referral of rural patients.

## Introduction

The modern movement of palliative care started in 1967 when Dame Cicely Saunders set up the first hospice, St. Christopher’s Hospice, in London. In the recent two decades palliative care has spread rapidly to continental Europe, America and other parts of the world [1], [2]. Owing to cultural, ethical, legal and other differences, utilization of palliative care varied among countries [2]. Even within a country, geographic variations might exist [3]–[5]. For example, hospice utilization in the United States has been reported to be higher in the South and Southwest and lower in the Midwest and Northeast [6]. Furthermore, within a region or district, people in remote areas might not have the same availability of hospice services as those in cities. A study in Canada showed the consistency and depth of palliative care service provision in British Columbia differed across community sites [7]. In India, the awareness of palliative care was known to be higher in urban areas than in rural areas [8]. The issue of urban-rural disparity thus deserves investigation in the development of palliative care worldwide. In Taiwan, all hospices are located in hospitals and with a higher probability in urban areas [9]. Such a hospital-based hospice system would perhaps widen the gap of palliative care use between urban and rural areas. The experience in Taiwan might contribute to the discussions of urban-rural disparity.

The aim of our study is to conduct a nationwide, population-based study to investigate the 7-year trend of hospice utilization in urban and rural areas in Taiwan. The analyses of urban-rural disparity not only focus on the resources and utilization of hospices but also the first-time choice of patients. The change of patient flows across the urban-rural border over time will shed more light on the development of hospice resources distribution.

## Materials and Methods

### Background

The first hospice ward in Taiwan was set up in 1987 [10]. The reimbursement of hospice ward care by the National Health Insurance in 2000 facilitated the flourishing development of hospices. Among all deaths, the proportion of patients receiving hospice care grew from 0.7% in 2000 to 4.81% in 2008 [11]. However, there is no independent hospice in the communities. All hospices in Taiwan are located in hospitals. In 2011, inpatient hospice service was provided by 49 hospitals with 723 beds available and home visits for palliative care were provided by 73 hospitals [12]. Most hospitals with an inpatient hospice unit were located in urbanized areas in Taiwan and only 6 hospitals with 80 hospice beds were in rural areas.

### Data Source

The data were obtained from the National Health Insurance Research Database (NHIRD) in Taiwan. The National Health Insurance started in 1995 and enrolled 99% of 23 million residents in Taiwan. The monthly claims by the contracted health care facilities are processed electronically and later aggregated to form the NHIRD for research use [13], [14]. The identification numbers of persons and health care facilities in the database have been encrypted to protect privacy, but the encrypted identification numbers remain unique so that record-linking within the database is feasible. All researchers who apply for use of the NHIRD are required to sign a written agreement declaring that they cannot violate the privacy of patients or health care providers and should acknowledge the NHIRD on publication.

Besides, the Taiwan Hospice Organization also provided the details of hospices and available beds in each year.

### Data Processing

At first, we identified patients who received inpatient hospice care from 1 July 2000 to 31 December 2006. Our analysis was limited to those aged > = 20 years at the time of their first admission to hospice ward. For each patient, we identified her/his first admission to hospice. The location of a hospice was converted into urban or rural type according to the definition of urbanization published by Taiwan’s National Health Research Institutes [15]. All 365 townships in Taiwan were classified into 7 clusters according to the following variables: population density (people/km^2^), population ratio of people with college or above educational levels, population ratio of elder people over 65 years old, population ratio of people of agriculture workers and the number of physicians per 100,000 people. In our current study, hospices located in clusters 1–3 were categorized as urban and the others as rural.

In the NHIRD, the information about each beneficiary’s residence is not available. A previous study [16] used the demographic characteristics, the type of beneficiaries and the location of medical visits for upper respiratory tract infection (URI) to estimate the residence of the population in NHIRD. The beneficiaries were legally classified into the following six categories [17]. Category 1 comprised civil servants in governmental agencies, employees of publicly or privately owned enterprises or institutions and employers or self-employed owners of business. Category 2 included the members of an occupational union and seamen serving on foreign vessels, who are members of the National Seamen’s Union or the Master Mariners’ Association. Category 3 was composed of farmers and fishers. Category 4 embraced military servicemen, military school students and those who are serving sentences in correctional institutions or receiving punishments from police and military court-martial. Category 5 was those members of a household of low-income families as defined by the Social Support Law. Category 6 included veterans and household representatives. This study found that the method which combined insurance classification, location of hospital visit, and insurance registration provided an optimal estimate of residence in each area by different levels of urbanization and age-group. To categorize the patients into urban and rural groups, we operationally devised an algorithm on the basis of the location of physician clinics in which the patients most frequently sought medical help due to URI (460–466 in International Classification of Diseases, Clinical Modification, version 9 [ICD-9-CM]) during 5 years before the first admission to hospice. We supposed that while cancer was normally treated at major hospitals the common URIs would be usually handled at physician clinics near a patient’s residence. If a patient sought medical help for URIs at urban clinics in more than or equal to 60% of such visits, she or he was deemed as an urban resident in our study. If a patient sought medical help for URIs at rural clinics in more than or equal to 60% of such visits, she or he was deemed as a rural resident. If none of the above conditions existed, the patient was deemed as a migratory resident.

Before 2009, the Taiwan’s National Health Insurance provided hospice care only for those patients with cancers and amyotrophic lateral sclerosis. The incidence of amyotrophic lateral sclerosis was much lower than cancers. Most patients receiving hospices care were terminal cancer patients. Therefore, our study population was limited to those patients with cancer diseases. The patient features under analysis included gender, monthly income, age and primary cancer diagnosis at the first hospice admission. A patient’s income could represent the socio-economic status. The diagnoses were grouped into most common cancers in Taiwan and western countries: lung/respiratory tract, hepatobillary tract, colon/rectal, pancreatic, esophageal, gastric, breast, prostate, urinary tract, hematologic-oncologic, head and neck, and others. Other diagnosis included other GI tract, bone, genital tract, brain, thyroid, other primary and secondary malignancy. We also investigate the location of the hospice chosen by a patient for the first time.

### Statistical Analysis

We used SPSS software (version 17) for statistical analysis in this study. The trend of hospice resources and the first-time admission to hospice in urban group and rural group were analyzed by Pearson’s χ2 tests. A *p* -value *<0.05* (two-tailed) was considered as statistically significant. For the demographic characteristics of cancer patients who received hospice care from 2000–2006, descriptive statistics were presented.

## Results

### Socio-demographic Characteristics

From 1 July 2000 to 31 December 2006, a total of 26,292 adult patients had been admitted to hospice in Taiwan (Table 1). There were more male patients than female (57.3% vs. 42.7%). Approximately two-fifths (42.6%) of patients were younger than 65 years old on their first-time admission. Three kinds of cancers, hepatocellular carcinoma, lung/respiratory tract cancer and colon/rectal cancer, accounted for almost a half of all cancer diagnoses. Three in four cancer patients receiving hospice care were urban residents. While lung/respiratory tract cancer was the most common diagnosis (19.3%) among urban patients, hepatocellular carcinoma ranked first (22.0%) among rural patients.

**Table 1:** Features of cancer patients with hospice care during 2000–2006, stratified by location of residence.

		Urban (%)	Rural (%)	Migratory (%)		Total (%)	
Total		19,689 (100)	6,037 (100)	566 (100)	26,292 (100)		
Gender	Female	8,547 (43.4)	2,411 (39.9)	263 (46.5)	11,221 (42.7)		
	Male	11,133 (56.5)	3,626 (60.1)	303 (53.5)	15,062 (57.3)		
Age	<65	8,664 (44.0)	2,293 (38.0)	232 (41.0)	11,189 (42.6)		
	65–74	4,989 (25.3)	1,925 (31.9)	168 (29.7)	7,082 (26.9)		
	75–84	4,854 (24.7)	1,522 (25.2)	132 (23.3)	6,508 (24.8)		
	>84	1,182 (6.0)	297 (4.9)	34 (6.0)	1,513 (5.8)		
Cancer diagnosis	Lung/respiratory	3,794 (19.3)	1,156 (19.1)	99 (17.5)	5,049 (19.2)		
	Hepatic	3,635 (18.5)	1,331 (22.0)	124 (21.9)	5,090 (19.4)		
	Colon/rectal	2,257 (11.5)	599 (9.9)	49 (8.7)	2,905 (11.0)		
	Head and neck	1,764 (9.0)	591 (9.8)	55 (9.7)	2,410 (9.2)		
	Gastric	1,405 (7.1)	348 (5.8)	46 (8.1)	1,799 (6.8)		
	Breast	989 (5.0)	194 (3.2)	21 (3.7)	1,204 (4.6)		
	Pancreatic	785 (4.0)	228 (3.8)	18 (3.1)	1,031 (3.9)		
	Urinary tract	697 (3.5)	244 (4.0)	23 (4.1)	964 (3.7)		
	Prostate	426 (2.2)	138 (2.3)	13 (2.3)	577 (2.2)		
	Hematological	415 (2.1)	130 (2.2)	13 (2.3)	558 (2.1)		
	Esophageal	488 (2.5)	187 (3.1)	17 (3.0)	692 (2.6)		
	Others	3,034 (15.4)	891 (14.8)	88 (15.5)	4,013 (15.3)		
Monthly income (Taiwan dollar)	< = 15,840	3,012 (15.3)	382 (6.3)	47 (8.3)	3,443 (13.1)		
	16,500–28,800	7,504 (38.1)	4,299 (71.2)	328 (58.0)	12,134 (46.2)		
	30,300–57,800	6,411 (32.6)	991 (16.4)	135 (23.9)	7,539 (28.7)		
	60,800–87,600	2,067 (10.5)	297 (4.9)	45 (8.0)	2,411 (9.2)		
	> = 92,100	686 (3.5)	68 (1.1)	11 (1.9)	765 (2.9)		

### Hospice Resources and Utilization

During the study period, the number of hospices increased from 16 with 305 beds to 34 with 557 beds (Table 2). Only 4 hospices were located in rural areas. The first rural hospice joined the program in 2002 with 23 beds. Although the number of beds in urban hospices increased from 305 to 492 during the 7 years, the proportion of rural hospice beds to all hospice beds rose from 5.8% in 2002 to 11.3% in 2006. The increasing trend of hospice beds was more marked in rural areas than in urban areas (p<0.001). Similarly, the utilization in rural hospices increased more significantly than that in urban hospices (p<0.001). The utilization of the first rural hospice was 3,041 patient-days (4.7% of all hospice utilization) in 2002. The utilization in 4 rural hospices rapidly expanded to 11,506 patient-days (11.6%) in 2006.

**Table 2:** Hospice resources and utilization from 2000–2006.

Year		2000	2001	2002	2003	2004	2005	2006	p-value
No. of hospices	Urban	16	20	23	24	25	28	30	<0.001
	Rural	–	–	1	1	2	3	4	
	Total	16	20	24	25	27	31	34	
No. of beds	Urban	305	356	391	414	434	468	492	<0.001
	Rural	–	–	23	23	31	53	65	
	Total	305	356	414	437	465	521	557	
Utilization(patient-days)	Urban hospice	28,418	60,884	60,997	58,888	71,584	78,987	87,610	<0.001
	Rural hospice	–	–	3,041	5,425	5,839	6,516	11,506	
	Total	28,418	60884	64038	64313	77423	85503	99116	

### Location of the Hospice Utilization

**Figure 1:**
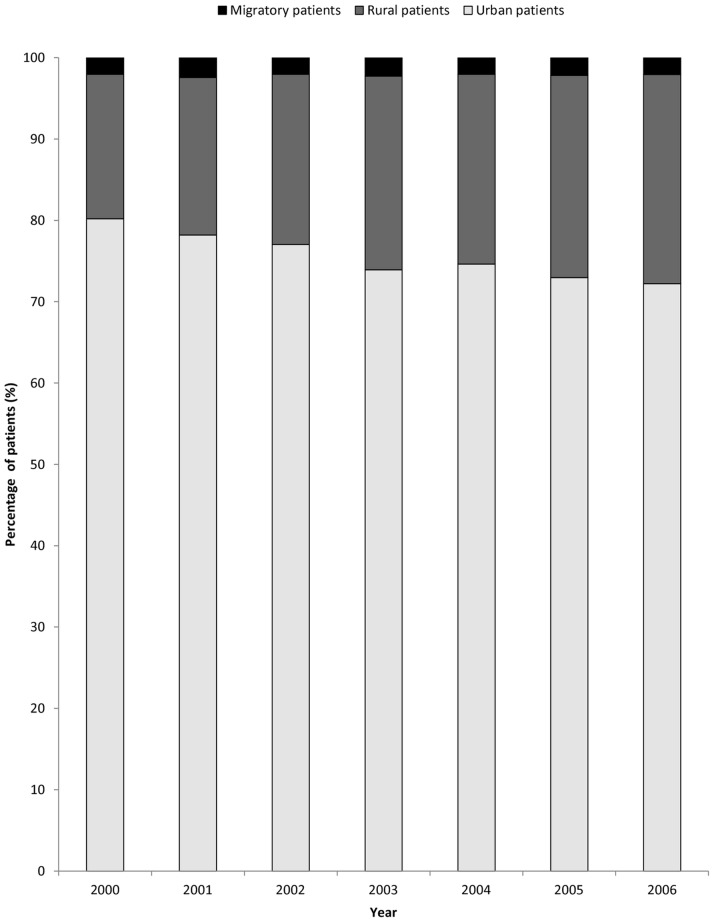
Urban-rural disparities of cancer patients with first-time hospice care from 2000–2006.

In the second half of 2000, a total of 1,706 patients had been admitted to hospice. The number of new hospice patients continuously increased to 5,277 in 2006 (Table 3). In the first two years (2000 and 2001), only two in ten patients receiving hospice care belonged to rural residents. The proportion of rural patients to all patients increased with time (Fig. 1, p<0.001) and reached 25.7% in 2006.

**Table 3:** The first-time admissions to hospices from 2000–2006, stratified by patient’s residence and hospice’s location.

		2000	2001	2002	2003	2004	2005	2006	Total
Urban patients	Urban hospice	1368	2480	2,769	2,684	3,023	3,177	3,609	19,110
	Rural hospice			46	112	116	103	202	579
	Total	1368	2480	2,815	2,796	3,139	3,280	3,811	19,689
Rural patients	Urban hospice	303	615	618	664	754	834	898	4,686
	Rural hospice			146	236	227	283	459	1,351
	Total	303	615	764	900	981	1,117	1,357	6,037
Migratory patients	Urban hospice	35	77	70	74	71	88	80	495
	Rural hospice			5	12	15	10	29	71
	Total	35	77	75	86	86	98	109	566
Total		1706	3172	3,654	3,782	4,206	4,495	5,277	26,292

In the first year when a rural hospice began to join the program, 19.1% of rural patients received hospice care for the first time at the rural hospice. The proportion increased to 33.8% in 2006 (Table 3). From the viewpoint of urban hospices, the percentage of rural patients fluctuated between 17% and 20% (17.8%, 19.4%, 17.9%, 19.4%, 19.6%, 20.3% and 19.5% from 2000 to 2006).

## Discussion

Although a hospital-based hospice system was adopted in Taiwan, the gap of palliative care use between urban and rural areas did not widen during the study period. While hospice utilization at both urban and rural areas increased with time, the annual percentage of rural patients in all hospice patients grew more significantly than that of urban patients. The growth of rural patients was largely attributed to the expanded capacity of rural hospices. However, at the end of the study period a substantial part of rural patients were still admitted to urban hospices.

The flourishing development of palliative care in Taiwan was facilitated by the government, through the enactment of Hospice Palliative Care Act in June 2000 [18] and the subsidy for hospital-based hospice services from the National Health Insurance in the same year [10]. The results in our study implied that the location of hospices played a major role of hospice utilization in rural areas. Similar development was also observed in Japan. Most palliative care units in Japan belong to general hospitals [19]. One study revealed that for most Japanese patients the inconvenient location of facilities could quite likely affect their willingness to receive palliative care [20]. In countries with vast territory such as the United States and Canada, the local provision of palliative care services might be more important in decreasing the urban-rural disparity. Studies had revealed that many patients in rural areas in the United States had poor access to hospice because of insufficient facilities, despite the Medicare Hospice Benefit [3], [21], [22]. Canada faced the similar problem [7], [23].

In our study, the growing percentage of rural patients in all hospices patients denoted either a rising demand of hospice services in rural areas or the delayed supply to rural areas in contrast to urban areas in the past years. After years of promotional measures in Taiwan, the existing cross-border use of hospices by rural patients implied that rural hospices satisfied only part of rural patients’ needs for hospice services. The supply of hospice services might be still insufficient in rural areas. The accessibility of hospice beds conditioned by geographical distance might play another role. For some rural patients, the traveling distance or time to neighboring villages with hospice services might be greater than that to cities [23]. The pattern of patient sources in hospices had been also reported to be an important factor. In rural Australia, most patients at community hospices were referred directly from hospitals [24]. In Taiwan, most hospice patients were referred from other wards of the same hospital [25]. The time of referral was usually close to death. According to an audit of a hospice at an academic medical center in Taiwan, 70% of cancer patients died in the hospice ward and only 4.9% were transferred to other institutions for further care [25]. That means if rural patients received cancer therapy at urban hospitals, they had little chance to be transferred to rural hospices. The consequences would be that rural patients were more likely to die at hospitals and most patients’ wishes to die at home [26], [27] could not be fulfilled. Finally, the patients’ preferences might influence their choice of location to receive hospice care. In the densely populated island country as Taiwan, the free choice of physicians without referral constraint made people to visit academic medical centers and metropolitan hospitals frequently [28]. Rural hospices still had to make efforts to enhance their images.

The role of reimbursements to hospitals for hospice care was less important in our analysis. The payment system of National Health Insurance in Taiwan started to include the hospice care (hospice ward and palliative home care) since July, 2000. During our study period, the regulations of reimbursements to hospice care didn’t change too greatly [29]. Some studies in the USA showed that hospice care was cheaper than other forms of end-of life care [30], [31]. In these studies, the cost represented total medical expense. However, the National Health Insurance in Taiwan covered most medical expenses not only for hospice care, but also for other hospital services. Patients didn’t need to pay less or more money when they received hospice care compared with other hospital service. Besides, hospice patients are also exempt from the usual copayments. Therefore, in such a medical environment in Taiwan, the effect of the payment system would be far less remarkable in our study.

There were some limitations in our study. The patients’ residences were operationally defined according to the location of physician clinics in which the patients most frequently sought medical help for upper respiratory tract infection in 5 years. The exact residences could not be validated because the NHIRD did not provide such information and not allow de-identification of patients and facilities for record linkage with external data sources, e.g. cancer registry or hospital records. Upper respiratory tract infection is indeed a very common cause for physician visits in Taiwan. A previous study revealed that diseases of the respiratory system accounted for one third of ambulatory visits within Taiwan’s National Health Insurance [28]. Because only a small percentage of patients could not be categorized as rural or urban group on the basis of the algorithm of our study, the bias of residence might be minimal. Another limitation of our study was that the trend comparison between rural and urban hospice patients was not undertaken with adjustment of related variables. Because the cases of terminal cancer could not be ascertained from the NHIRD and other detailed information of each terminal patient could not be obtained, the advanced statistical analysis was not feasible. Besides, the changes of health care environment in the meantime, e.g. other policy changes of the NHI, the consolidation of large hospitals and increased hospital competition in the meantime, might also influence the urban-rural disparities in hospice utilization. Finally, the observation of our study might be late and short. The first hospice in Taiwan was set up 13 years earlier. The development of hospices expanded from urban to rural areas. We might have observed only the catch-up phase of rural areas. Perhaps after some years the urban-rural disparity would become clearer. However, since 2009 the hospice services within Taiwan’s National Health Insurance had been extended to terminal patients of non-cancer diseases, i.e. dementia, congestive heart failure, chronic renal disease, chronic hepatic disease, chronic obstructive pulmonary disease and acquired immunodeficiency syndrome. The heterogeneity of patients would make future analyses more complicated and difficult.

### Conclusions

There is a growing trend of resources and utilization in both urban and rural areas in Taiwan. Although rural hospices developed more markedly than urban hospices during the study period, a substantial part of rural patients still received care across the urban-rural border. Further studies can be devoted to analyzing whether insufficient supply of rural hospices or inefficient referral of rural patients exists. The expanding provision of non-institutional services, e.g. home visit, may be also an option to increase the accessibility of palliative care in rural areas.
